# Assessing and Reporting Household Chemicals as a Novel Tool to Mitigate Pesticide Residues in Spinach (*Spinacia oleracea*)

**DOI:** 10.1038/s41598-018-37936-2

**Published:** 2019-02-04

**Authors:** Rai Muhammad Amir, Muhammad Atif Randhawa, Muhammad Nadeem, Anwaar Ahmed, Asif Ahmad, Moazzam Rafiq Khan, Muhammad Asif Khan, Rukhsana Kausar

**Affiliations:** 1 Institute of Food and Nutritional Sciences, PMAS-Arid Agriculture University, Rawalpindi, Pakistan; 2 National Institute of Food Science and Technology, University of Agriculture, Faisalabad, Pakistan; 3 Department of Environmental Sciences, COMSATS University Islamabad, Vehari Campus, Pakistan; 4 University of Agriculture Faisalabad, Sub-campus, Burewala/Vehari, Pakistan; 5 Department of Environmental Science, International Islamic University, Islamabad, Pakistan

## Abstract

Non-selective use of pesticide to increase productivity leaves residues on crops. These pesticides after consumption have a detrimental effect on human health and their intake can lead to various diseases such as kidney damage, neurological disorders, cardiovascular diseases, circulatory and reproductive problems. The recent study was designed to assess the effects of household processing treatments such as acidic solutions (acidic acid and citric acid), alkaline solutions (sodium chloride and sodium carbonate) and biological solutions (radish, ginger, garlic, and lemon extracts) were evaluated for their efficiency for removal of pesticides in spinach by gas chromatography with electron capture detector (GC-ECD). The results showed that pesticide residues were sharply reduced when spinach was exposed to washing treatments. The results showed that the greatest reduction of deltamethrin, cypermethrin, chlorpyrifos and endosulfan with 10% acetic acid was (79.68 ± 0.81%), (89.99 ± 0.12%), (94.21 ± 0.02%) and (70.32 ± 0.25%), respectively of tested washing solutions. The acidic solutions were more effective than alkaline and biological extracts in the removal of pesticide residues. The average reduction in various washing solutions ranged from 10.21 to 79.68%, 22.60 to 89.48%, 22.95 to 94-21% and 11.24–70.32% for the removal of deltamethrin, cypermethrin, chlorpyrifos, and endosulfan, respectively.

## Introduction

Vegetables are delicious parts of a herbaceous plants that are believed to be natural container of key nutrients worldwide, which plays a vital role in food security. They are the basic nutrition and are extremely supportive of human capacity by providing nutrients such as calcium, magnesium and iron, dietary fiber, antioxidants, micronutrients and vitamins like A, C, and B complex^1,2^. Furthermore, vegetables consumption plays a key role in the prevention of various diseases, repair, maintenance and building the body organs^3^. Spinach (*Spinacia oleracea*) usually consumed vegetable around the world belongs to the Amaranthaceae family. In developing countries such as Pakistan, it is obligatory to spray vegetables to get higher yield, farmers practice pesticides during the growth season even at a mature stage and ignoring the proposed period between the latest spraying and harvest. The application of pesticide accumulates in to internal parts of vegetables and eventually pesticides are found to be a residues that causing serious illness to the consumer^4,5^.

Organochlorine pesticides are very slowly disintegrated and its display at very low concentration over a long period can ultimately lead to serious health hazards to the organism. They cause a neurological problem in interaction with the brain associated acetylcholinesterase enzyme. Organophosphates pesticides cause reproductive issues by acting on human reproductive enzymes and various pests prevented by synthetic pyrethroids due to their wide range of insecticidal activities, rapid biotransformation and their non-adherence to the environment. Pesticide residues have become a part of the food chain just because of of inadequate and excessive use on the foods and they interfere with the cellular respirator and their long term exposure causes skin problems, anorexia and damage immune system^6,7^.

The tap water washing proves a vital technique for the mitigation of pesticide residues in various vegetables such as tomato, okra, brinjal, cauliflower and potatoes. The level of endosulfan residues increased during the uncooked phase of tomato and potatoes than in the cooking phase. By using household washing techniques,residues of endosulfan decreased drastically below maximum residues limits (MRLs)^8^. It was shown that the biological reddish extract was more effective in reducing organochlorine pesticides while acidic and alkaline washing solutions proved more effective in reducing organophosphate residues than organochlorine in potatoes^9^.

Vegetables soaking with different acidic solutions (hydrogen peroxide, acetic acid, citric acid, and ascorbic acid) proved to be an effective tool for reducing pesticide residues. Simple washing can be used to remove residues of pesticide and dominantly uses sodium chloride to mitigate residues of fruits and vegetables. However, blanching and washing with hot water is more effective than cold water washing^10^. Washing with a detergent solution proved to be very effective in taking way pesticides residues and for twenty minutes of immersion in a detergent solution reduced pesticide residues from 31.1% to 98.8%. A 5% Sodium bicarbonate solutions significantly removes the residues of chlorpyrifos and fenitrothion in different vegetables whereas ultrasonic cleaning decreases pesticide residues ranging from 49.8 to 84.4% at 40 C for 48 hr^11^. Potato dipping in 5% acetic acid solution decreases 69%, 10% solution reduces 95%, Tap water 15%, sodium chloride solution 5% reduces 35% and 10% mitigates 50.6% organochlorine residues^12^.

Regular consumption pattern of contaminated foods contributed significantly in the deposition of pesticide residues in the body and their adverse effects become prominent after several years of exposure. Consistent examination and review of these pesticide residues in foodstuff is very essential to prevent the contamination of food chain from these toxicants. Due to the cumulative behavior, persistent nature and their toxic potential effects, it is the basic necessity to monitor and test this type of toxicants and to determine the most effective way to get rid of these contaminants in food supply chain especially in spinach. So, the recent study has been designed to find out the pesticide residues in spinach that will be procured from self-grown field and residues will be compared with their corresponding maximum residues limits (MRLs) and elucidate the effectiveness of different washing techniques like alkaline, chemical, and biological solutions to minimize the pesticide residues. Currently, there is limited data available regarding mitigating the impact of pesticide residues in vegetables not in Pakistan but throughout the world. Therefore, the present project has been planned to have a deep insight into the problem and to find out suitable solutions to combat the issue.

## Materials and Methods

### Collection of vegetable samples

The spinach was harvested from self-grown, supervised field at optimum maturity after 36 h wait for foliar insecticide spray in the absence of air and precipitation. A known amount of of different pesticides such as deltamethrin, cypermethrin, chlorpyrifos, and endosulfan has been applied. One kilogram of spinach was obtained at the point of optimum maturity. Samples were immediately transferred to the laboratory of National Institute of Food Science and Technology, for subsequent analysis. Pesticides standards were purchased from Dr. Ehrenstrofer GmbH (Augsburg, Germany).

### Soaking of spinach in washing solutions

The collected spinach samples were soaked with different acidic, alkaline and biological extracts of different concentrations along with tap water for the comparison purpose (Table 1). The samples were soaked in washing solutions for 10 min at 30 °C. Subsequently, the samples were reduced to the appropriate size using the knife to facilitate analysis and blended using a domestic National blender (MJ176NR) to obtained a homogeneous suspenion^9^.

**Table 1:** Treatment plan for mitigation of pesticide residues in spinach.

Treatments	Concentrations (%)				
T_0_ Unwashed Samples	—	—	—	—	—
T_1_ Tap water washing	—	—	—	—	—
T_2_ Acetic acid	2	4	6	8	10
T_3_ Citric acid	2	4	6	8	10
T_4_ Sodium chloride	2	4	6	8	10
T_5_ Sodium carbonate	2	4	6	8	10
T_6_ Ginger extract	2	4	6	8	10
T_7_ Garlic extract	2	4	6	8	10
T_8_ Radish extract	2	4	6	8	10
T_9_ Lemon extract	2	4	6	8	10

### Extraction of pesticide residues

In spinach sample, pesticide residues were extracted by using the method of Khan *et al*.^13^. During this extraction method ethyl acetate was used as a solvent medium for its significant recovery purpose. The homogenized spinach sample (50 g) was placed in 250 ml Erlenmeyer flask, 20 g of anhydrous sodium sulfate was added and thoroughly mixed for homogenously mixing to avoid clod formation. After uniform mixing,10 ml of saturated sodium chloride and 75 ml of ethyl acetate were added together with glass beads to facilitate the extraction process. The flask containing the mixture was placed on a horizontal mechanical shaker at 240 rpm for a period of 1 hour. After shaking, the extract was procured in a plastic bottle and filtered using Whatman (No. 4) filter paper. The filtered extract was stored at 40 C for further analysis.

### Clean-up of filtered extract

Pesticide residues are traceable and require high sensitivity. In order to achieve a high level of sensitivity, a clean-up operation was used to remove color and other impurities for precise determination of pesticide residues. Accordingly, various residues of pesticide were cleaned by column chromatographic method^14^. During this process, glass wool was placed on the bottom of the column with a thin layer of anhydrous sodium sulfate placed on glass wool. The addition of activated charcoal and silica gel mixture in a ratio of 7:5 (w/w) were placed on a sodium sulfate layer. Charcoal and silica gel were activated at 200 C for 48 hours before column filling. Furthermore, the activated charcoal and silica gel mixture were covered with a thin layer of anhydrous sodium sulfate and glass wool. Column clean-up is now ready to clean the extracted sample and adjust the flow rate 1 ml per minute before loading the required sample.

The sample extract after column preparation and extraction was eluted with 50 ml of hexane and acetone mixture (7:3 v/v) and clean-up was eluted in a 150 ml plastic bottle. The rotary evaporator was used to concentrate the elute at 40 °C up to 1 to 1.5 ml. Glass sucker was used to transfer concentrated elute into vials of volume 1.5 ml and the elute was completely dried under the mild steam of nitrogen.

### Quantification of pesticide residues through Gas Chromatography

GC-ECD was used to determine pesticide residues in the spinach sample as illustrated by Sannino *et al*.^15^, and Steel *et al*.^16^. The parameters were set as inlet temperature 280 °C, detector temperature 300 °C, filtered prepared sample was 2 µL and nitrogen gas was used as carrier. The total flow rate was set at 60 ml/min and column flow rate was 2 ml/min.

### Statistical Analysis

The data obtained after three independent replicates were analyzed by completely randomized design using standard statistical procedures (Statistix 8.1). Moreover, Analysis of Variance (ANOVA) was performed to determine the level of significance while the least significant difference (LSD) test was used to compare the means of treatments^17^.

## Results

The results pertaining pesticide residues reduction in spinach has been shown in Table 2. These results show that the combination of different treatments and concentration predominantly alleviate the pesticide residues. Likewise, it was discovered that subtraction of pesticide residues was strongly influenced by combination of treatments and concentration with their interaction. Pesticide residues showed a linear reduction pattern in the spinach.

**Table 2:** Analysis of variance for pesticide residues reduction in spinach.

Source of variations	Degrees of freedom	Mean squares			
		Deltamethrin	Cypermethrin	Chlorpyrifos	Endosulfan
Combinations	40	1533.9**	1214.6**	1551.79**	1059.7**
C vs others	1	3901.9**	2321.4**	4029.86**	2509.1**
Treatment (T)	7	194.0**	655.4**	159.76**	254.3**
Conc. (C)	4	13913.9**	10182.5**	14189.88**	9371.9**
Linear	1	54314.1**	39848.7**	5440.37**	36309.2**
Quadratic	1	1106.7**	669.1**	1170.53**	900.4**
Cubic	1	143.6**	150.8**	128.84**	194.9**
Quartic	1	91.4**	61.3**	75.80**	83.4**
T x C	28	15.7**	33.8**	8.55**	21.8**
Error	82	0.7	1.3	0.49	1.2
Total	122				

NS = Non-significant (P > 0.05); *Significant (P < 0.05); **Highly significant (P < 0.01).

Results related to the interaction with treatment and concentration are shown in Table 3. Deltamethrin residues have been significantly reduced with the linear increase in treatments concentration. Among the acid washing treatments, the percentage reduction of deltamethrin was in the range of 11.67 ± 0.28–79.68 ± 0.81%. Spinach treated with acetic acid (10%) showed a maximum decrease of 79.68 ± 0.81% while citric acid (2%) recorded the lowest reduction of 11.67 ± 0.28%. In the case of an alkaline solution, the percent reduction of residues was in the range of 11.79 ± 0.54–73.51 ± 0.35%. Sodium carbonate (10%) showed a maximum reduction of 73.51 ± 0.35% and sodium chloride (2%) exhibited minimal reduction of 11.79 ± 0.54%. Among the biological extracts, the percent reduction of deltamethrin was in the range of 10.21 ± 0.58–69.81 ± 0.56%. The reddish extract (2%) showed minimal decrease of 10.21 ± 0.58% while the lemon extract (10%) gave a maximum reduction of 69.81 ± 0.56%. The total data shown in Table 3 revealed that the spinach treated with acetic acid (10%) exhibited a maximum reduction of residues of deltamethrin.

**Table 3:** Interactive effects of treatments on reduction of deltamethrin residues in spinach.

Treatment	Concentration				
	2%	4%	6%	8%	10%
Acetic Acid	12.12 ± 0.22t	32.21 ± 0.24p	54.55 ± 0.30k	68.46 ± 0.42de	79.68 ± 0.81a
Citric Acid	11.67 ± 0.28t	29.63 ± 0.63pq	50.96 ± 0.50 l	65.55 ± 0.23f	74.52 ± 0.32b
Sodium Chloride	11.79 ± 0.54t	28.62 ± 0.53qr	50.51 ± 0.29 l	62.18 ± 0.22gh	70.93 ± 0.53cd
Sodium carbonate	12.12 ± 0.22t	29.85 ± 0.74pq	51.18 ± 0.59 l	64.87 ± 0.34fg	73.51 ± 0.35bc
Ginger extract	11.34 ± 0.32t	29.07 ± 0.37qr	49.72 ± 0.55lm	61.62 ± 0.43hi	67.56 ± 0.53ef
Garlic extract	11.00 ± 0.40t	26.49 ± 0.85rs	45.91 ± 0.39n	58.03 ± 0.54j	65.10 ± 0.34f
Reddish extract	10.21 ± 0.58t	23.79 ± 0.42s	42.87 ± 0.62o	51.40 ± 0.46l	59.37 ± 0.35ij
Lemon extract	11.22 ± 0.45t	27.83 ± 0.82qr	47.70 ± 0.58mn	60.83 ± 0.41hi	69.81 ± 0.56de

Means sharing the similar letter in a row or in a column are statistically non-significant (P > 0.05). Small letters represent comparison among interaction means.

Moreover, the results with respect to cypermethrin are depicted in Table 4 and this study showed, by increasing the chemical concentration in the solution results also reduces the the amount of residues. Among acid treatments, cypermethrin residues were found in the range of 27.80 ± 0.62–89.99 ± 0.12%. The maximum percent reduction in cypermethrin residues (89.99 ± 0.12%) was recorded when 10% acetic acid was applied to spinach and the minimum residual reduction (27.80 ± 0.62%) was observed when 2% citric acid was applied. Among salt solutions, residues of cypermethrin were present in the range of 23.88 ± 0.60–80.48 ± 1.31%. Sodium carbonate (10%) showed a maximum reduction of 80.48 ± 1.31% and sodium chloride (2%) expressed minimum reduction potential in the elimination of residual cypermethrin. In the case of biological extracts, the spinach treated with 10% ginger extract gave up the highest reduction in cypermethrin (73.04 ± 0.12%) followed by lemon extract (68.68 ± 0.36%), garlic extract (65.89 ± 0.44%) and reddish extract (60.68 ± 1.37%). The lowest reduction (22.60 ± 0.51%) was observed when 2% reddish extract was applied to spinach. In conclusion, the results showed that acetic acid (10%) indicated the greatest reduction in cypermethrin.

**Table 4:** Interactive effects of treatments on reduction of cypermethrin residues in spinach.

Treatment	Concentration				
	2%	4%	6%	8%	10%
Acetic Acid	31.88 ± 0.82s	47.04 ± 0.33lm	67.79 ± 0.11ef	80.48 ± 0.59b	89.99 ± 0.12a
Citric Acid	27.80 ± 0.62t	42.23 ± 0.23no	64.37 ± 0.09fgh	77.13 ± 0.34b	86.63 ± 0.18a
Sodium Chloride	23.88 ± 0.60u	39.71 ± 0.62op	58.78 ± 0.63i	71.76 ± 0.30cd	78.24 ± 0.86b
Sodium carbonate	24.67 ± 0.64tu	40.61 ± 0.32op	61.07 ± 0.30hi	73.21 ± 0.40c	80.48 ± 1.31b
Ginger extract	26.12 ± 0.62tu	38.03 ± 1.07pq	54.70 ± 0.33j	65.21 ± 0.64efg	73.04 ± 0.12c
Garlic extract	22.60 ± 0.51u	35.29 ± 0.20qrs	50.73 ± 0.56kl	60.79 ± 0.59hi	65.89 ± 0.44efg
Reddish extract	22.60 ± 0.51u	33.89 ± 0.24rs	45.75 ± 1.62mn	54.42 ± 0.90jk	60.68 ± 1.37hi
Lemon extract	24.44 ± 0.52tu	37.47 ± 0.52pqr	53.41 ± 1.28jk	62.81 ± 0.34gh	68.68 ± 0.36de

Means sharing the similar letter in a row or in a column are statistically non-significant (P > 0.05). Small letters represent comparison among interaction means.

In the case of chlorpyrifos, it was detected that the minimum confiscation of chlorpyrifos residues (22.95 ± 0.02%) was found when 2% of reddish extract was applied to spinach while 10% acetic acid showed maximum reduction of chlorpyrifos (94.21 ± 0.02%) followed by citric acid (90.54 ± 0.04%), sodium carbonate (86.40 ± 0.04%), sodium chloride (79.33 ± 0.03%), ginger extract (75.20 ± 0.01%), lemon extract (71.25 ± 0.04%), garlic extract (68.50 ± 0.04%) and reddish extract (65.38 ± 0.04). Conclusively, among all washing treatments acetic acid (10%) gave up the highest reduction in chlorpyrifos in the spinach as shown in Table 5.

**Table 5:** Interactive effects of treatments on reduction of chlorpyrifos residues in spinach.

Treatment	Concentration				
	2%	4%	6%	8%	10%
Acetic Acid	31.68 ± 0.01w	48.39 ± 0.03p	71.99 ± 0.01g	85.95 ± 0.02c	94.21 ± 0.02a
Citric Acid	29.84 ± 0.06x	44.62 ± 0.05q	69.23 ± 0.01h	82.64 ± 0.02d	90.54 ± 0.04b
Sodium Chloride	26.17 ± 0.01z	40.22 ± 0.03l	58.95 ± 0.02l	69.05 ± 0.03h	79.33 ± 0.03e
Sodium carbonate	27.82 ± 0.02y	41.87 ± 0.02r	66.48 ± 0.01i	79.70 ± 0.01e	86.40 ± 0.04c
Ginger extract	24.60 ± 0.03a	37.46 ± 0.03t	56.47 ± 0.01m	65.38 ± 0.03i	75.20 ± 0.01f
Garlic extract	23.32 ± 0.05ab	34.43 ± 0.03uv	50.96 ± 0.01o	60.78 ± 0.03k	68.50 ± 0.04h
Reddish extract	22.95 ± 0.02b	33.42 ± 0.01v	47.93 ± 0.05p	58.03 ± 0.03l	65.38 ± 0.04i
Lemon extract	23.69 ± 0.02ab	35.53 ± 0.03u	53.71 ± 0.01n	62.62 ± 0.03j	71.25 ± 0.04g

Means sharing the similar letter in a row or in a column are statistically non-significant (P > 0.05). Small letters represent comparison among interaction means.

The results related to endosulfan are shown in Table 6, which revealed that increasing the concentration of the washing agent also increases the rate of reduction of residues. Among acid washing treatments, the percent reduction of endosulfan ranged from 12.59 ± 0.78–70.32 ± 0.25%. The spinach treated with 10% acetic acid showed maximum decrease of endosulphan residues as 70.32 ± 0.25% while minimal residue reduction (12.59 ± 0.78%) was found when 2% citric acid was applied. In alkaline washing treatments, 10% sodium carbonate abandoned the residual reduction (65.60 ± 0.25%) while 2% sodium chloride was the lowest residue reduction (11.35 ± 0.75%). In the case of biological extracts, the percent reduction of endosulfan was in the range of 11.24 ± 0.95–56.66 ± 0.38%. Ginger extract (10%) showed the highest percent decrease (56.66 ± 0.38%) while reddish extract (2%) causes the lowest reduction of endosulfan residues (11.24 ± 0.95%). Overall, acetic acid proved to be more effective in mitigating the pesticide residues in spinach due to its greater reduction power.

**Table 6:** Interactive effects of treatments on reduction of endosulfan residues in spinach.

Treatment	Concentration				
	2%	4%	6%	8%	10%
Acetic Acid	13.37 ± 0.50q	30.47 ± 0.14m	51.10 ± 0.29fgh	65.26 ± 0.18b	70.32 ± 0.25a
Citric Acid	12.59 ± 0.78q	28.05 ± 2.05mn	46.99 ± 0.30ij	58.06 ± 0.21c	67.12 ± 0.26ab
Sodium Chloride	11.35 ± 0.75q	24.45 ± 0.87nop	40.08 ± 0.22l	51.21 ± 0.56fgh	57.51 ± 0.36c
Sodium carbonate	12.53 ± 1.17q	26.64 ± 0.59no	46.37 ± 0.86jk	57.05 ± 0.80cd	65.60 ± 0.25b
Ginger extract	11.97 ± 0.69q	24.05 ± 0.49op	42.72 ± 0.63kl	51.60 ± 0.51efg	56.66 ± 0.38cd
Garlic extract	11.58 ± 0.32q	23.50 ± 0.22op	40.25 ± 0.31l	49.18 ± 0.86g-j	53.52 ± 0.42def
Reddish extract	11.24 ± 0.95q	22.76 ± 0.69p	39.57 ± 0.63l	47.78 ± 0.12hij	50.14 ± 0.41f-i
Lemon extract	12.03 ± 0.75q	23.89 ± 0.54op	40.47 ± 0.70l	49.30 ± 0.06g-j	54.97 ± 0.38cde

Means sharing the similar letter in a row or in a column are statistically non-significant (P > 0.05). Small letters represent comparison among interaction means.

## Discussion

According to data obtained during recent study, the remains of various pesticides are drastically reduced when spinach was dipped into different washing solutions. The results showed that the solutions used for soaking have an effective role in the alleviation of toxic residues. The organophosphate (chlorpyrifos) was degraded more rapidly followed by pyrethroids (deltamethrin and cypermethrin) and the lowest decrease was recorded in organochlorine (endosulfan).

Organophosphate and pyrethroids showed more ability to reduce their residues due to their dissolution power and unique physiochemical properties compared to the organochloride. The organochloride has a specific ability to retain in the soil crust, different parts of plants and animal tissues due to the long biological half-lives. Spinach samples collected from supervised field trial found the residues beyond the MRLs but by soaking in various washing treatments they have an effective role in removing pesticide residues. Vegetables treating with simple water washing alleviates most of the pesticide residues that are held on the surface of the vegetables, which can cause the residues of pesticides in vegetables to be subjected to washing treatments.

Differences in mitigation of pesticide residues during the washing process have been found in study that may be due to differences in the nature and behavior of chemicals. From acidic solutions, acetic acid has shown a strong attitude in removing pesticide residues as compared to citric acid because acetic acid has more power as a chelating agent which makes the residues of pesticides unavailable. In the case of a salt solution, sodium carbonate showed a greater reduction power compared to sodium chloride because it was combine with simple water to remove toxic residues. Amongst biological extracts, the ginger extract played an effective role in removing toxic residues as compared to other biological extracts because it is equipped with the active ingredient gingerol that binds pesticide residues. Consequently, among all washing treatments, it has been found that the acidic detergent is most effective for the reduction of organophosphates, pyrethroids and organochlorine from alkaline and biological extracts.

The results of the current study showed additional support from previous findings of Zohair^9^, who found that pesticide residues were drastically removed when brinjal and potato were carried out. These results also have similarities with the earlier findings of Soliman^18^, who found that tap water reduced a significant amount of various pesticide residues in vegetables. Similar results have been shown from the investigations of Kaushik *et al*.^19^, who exhibited potato dipping in 5% acetic acid solution reduce 69%, 10% mitigate 95%, Tape water 15%, sodium chloride solution 5% reduce 35% and 10% mitigate 50.6% residues of organochlorine.

The present results are also in parallel with the earlier finding described by Randhawa *et al*.^8^, who determined that peel reduced deltamethrin residues from 76–80%, simple washing mitigate 10–35% and heat treatment removed 19–40% in all analyzed vegetables. These results have also a strong agreement for the mitigation of pesticide residues from vegetables with the earlier finding of Abdullah *et al*.^20^. The finding regarding the decline in pesticide residues through various washing treatments are harmonized with Randhawa *et al*.^8^ and Liang *et al*.^11^ who revealed a similar reduction in pesticide residues.

## Conclusions

This study has demonstrated that mitigating the risk associated with indiscriminate use of pesticides, soaking the spinach in different washing treatments such as acetic acid, citric acid, sodium chloride, sodium carbonate, ginger, garlic, lemon and reddish extract can be an effective tool for minimizing pesticide residues. Generally, pesticide residues have been significantly reduced with linear increase in treatment concentrations. Among acidic solutions, acetic acid has shown a strong attitude towards removal of pesticide residues compared to citric acid and salt solution, sodium carbonate exhibited more reduction power compared to sodium chloride while among the biological extracts,ginger extract played an effective role in removing poisonous residues compared to other biological extracts. Consequently, among all washing treatments, acetic acid (10%) was proved most effective for mitigating the residues of organophosphates, pyrethroids and organochlorine than alkaline and biological extracts. So, at the household level, the vegetables must be washed with tap water as well as household chemicals to eliminate the residues of contaminants.
